# Memory rehabilitation: restorative, specific knowledge acquisition, compensatory, and holistic approaches

**DOI:** 10.1007/s10339-022-01099-w

**Published:** 2022-07-05

**Authors:** Yashoda Gopi, Edward Wilding, Christopher R. Madan

**Affiliations:** 1 School of Psychology, University of Nottingham, Nottingham, NG7 2RD UK; 2 School of Psychology, University of Birmingham, Birmingham, UK

**Keywords:** Memory impairment, Memory rehabilitation, Acquired brain injury, Memory strategies, Memory aids

## Abstract

Memory impairment following an acquired brain injury can negatively impact daily living and quality of life—but can be reduced by memory rehabilitation. Here, we review the literature on four approaches for memory rehabilitation and their associated strategies: (1) the restorative approach, aimed at a return to pre-morbid functioning, (2) the knowledge acquisition approach, involving training on specific information relevant to daily life, (3) the compensatory approach, targeted at improving daily functioning, and (4) the holistic approach, in which social, emotional, and behavioral deficits are addressed alongside cognitive consequences of acquired brain injury. Each memory rehabilitation approach includes specific strategies such as drill and practice (restorative), spaced retrieval (knowledge acquisition), memory aids (compensatory), or a combination of psychotherapy and cognitive strategies (holistic). Past research has demonstrated mixed support for the use of restorative strategies to improve memory function, whereas knowledge acquisition strategies show promising results on trained tasks but little generalization to untrained tasks and activities of daily living. Compensatory strategies remain widely used but require intensive training to be effectively employed. Finally, the holistic approach is becoming more widespread due to improvements in psychosocial wellbeing, yet there are considerable resource and cost requirements. Several factors can influence rehabilitation outcomes including metacognition and emotional disturbances. Considerations for future research to improve the applicability of strategies for memory rehabilitation include assessing memory impairment severity, examining memory needs in daily life, and exploring the long-term effects of memory rehabilitation.

## Introduction

An acquired brain injury (ABI) involves an insult to the brain resulting in neuronal activity changes and can be derived from traumatic brain injuries (TBI) such as accidents or falls, or from non-traumatic internal events such as stroke (De Luca, et al. 2018a, b; Fernandez et al. 2017; Rees et al. 2007; Turner-Stokes et al. 2003). Memory impairment following an ABI can negatively impact daily living and quality of life (Claesson et al. 2005; Pohjasvaara et al. 1997; Slenders et al. 2020; Tang et al. 2020; Wood 2017). Moreover, previous research has indicated that there is potentially a link between ABI and progression to dementia; however, the methodologies of previous studies have been mixed or of poor quality and findings should be considered with caution (das Nair et al. 2016; Hicks et al. 2019; Kuźma et al. 2018). Memory deficits are among the most common cognitive deficits and subjective complaints for stroke and TBI survivors in hospitals, rehabilitation centers, and community settings (Barman et al. 2016; Censori et al. 1996; Cumming et al. 2013; das Nair et al. 2016; Hochstenbach et al. 1998; Shigaki et al. 2014; Wade et al. 1986). Although some improvements in memory abilities can occur through spontaneous recovery, memory impairments can persist for years after the injury (Censori et al. 1996; Elliott and Parente 2014; Galetto and Sacco 2017; McInnes et al. 2017; Rasquin et al. 2002; Schaapsmeerders et al. 2013; Zucchella et al. 2014). Cognitive rehabilitation is one option to reduce the impact of memory impairment.

Cognitive rehabilitation involves the use of various techniques expected to improve functioning in one or more cognitive domains (De Luca, et al. 2018a, b; Galetto and Sacco 2017; Lincoln et al. 2002; Rees et al. 2007; Wilson 2013). The techniques are used to restore function, to provide specific information relevant to everyday life, and as supportive tools to improve daily functioning (Cumming et al. 2013; das Nair et al. 2016; das Nair and Lincoln 2012; Rees et al. 2007; Schacter and Glisky 1986; Shigaki et al. 2014). Cognitive rehabilitation that is intended to improve memory is commonly thought to involve four approaches (das Nair et al. 2016; das Nair and Lincoln 2012; Prigatano 2013; Rees et al. 2007; Tate 1997; Wilson 2009). The *restorative approach* involves a re-training of memory abilities, the *specific knowledge acquisition approach* involves learning information relevant to a particular need in everyday life, and the *compensatory approach* involves the use of internal and external memory aids for various situations (Cicerone et al. 2000; Pertíñez and Linares 2015; Pino 2015; Schacter and Glisky 1986; Tsaousides and Gordon 2009). More recently, a *holistic approach* has emerged whereby emotional and social factors are addressed alongside cognitive consequences of ABI using a combination of compensatory aids and a therapeutic component (Ben-Yishay 2000; Cicerone et al. 2019; Prigatano 2013).

Practice guidelines and recommendations for implementation of evidence-based cognitive rehabilitation within clinical settings can be found in dedicated manuals (Haskins et al. 2012; Intercollegiate Stroke Working Party 2016; van Heugten and Wilson 2021; Velikonja et al. 2014). In this review, we consider each of the outlined memory rehabilitation approaches, focusing on their implementation with stroke and TBI survivors. Although similar reviews have been conducted in the literature, they have included a variety of patient groups, examined general cognitive rehabilitation inclusive of other cognitive domains, or have focused on specific strategies within an approach (Barman et al. 2016; Clare and Jones 2008; Pino 2015; Shigaki et al. 2014; Tate 1997; Tsaousides and Gordon 2009). While these subjects are worth examining, it would be beneficial to have an overview of the rehabilitation approaches available to target memory deficits and the strategies that are currently in use for stroke and TBI patients. Here, we specifically outline the major approaches to memory rehabilitation, explore the strengths and limitations of strategies within each approach, highlight factors that may influence the outcome of rehabilitation, examine limitations of current approaches, and provide considerations for researchers and clinicians regarding memory rehabilitation after ABI.

## Restorative approach

The goal of the restorative approach (also referred to as *retraining* or *restitution)* is to improve memory to a level similar to that of pre-morbid functioning (De Luca, et al. 2018a, b; Pino 2015; Prigatano 1987; Rothi and Horner 1983; Schacter and Glisky 1986; Velikonja et al. 2014). It is widely held that brain plasticity underlies the restorative approach and that activation of the residual neural network surrounding the damaged brain tissue may at least partially improve functional outcomes and prevent further loss (Berlucchi 2011; Cumming et al. 2013; Kleim and Jones 2008; Rothi and Horner 1983; Zencius et al. 1990). Although it is impossible for damaged neurons to be replaced or regenerated, it is considered possible that surviving neurons can establish new connections through axonal and dendritic sprouting and synaptogenesis (Berlucchi 2011; Kane and Ward 2021; Murphy and Corbett 2009). This should in turn result in plasticity-dependent remodeling of the neural networks where connectivity patterns may be altered following the presentation of new stimuli (Berlucchi 2011; Caeyenberghs et al. 2018; Gabrieli et al. 2021; Galetto and Sacco 2017). Strategies falling within the restorative approach have traditionally centered around repetitive drill and practice exercises, stemming from the idea that the brain is a “mental muscle” that can be strengthened through exercise when damaged (das Nair et al. 2016; das Nair and Lincoln 2012; Harris and Sunderland 1981; Schacter and Glisky 1986; Tate 1997). More recently, there has been a shift to computer-assisted cognitive rehabilitation (CACR) based on limited efficacy of drill and practice (De Luca, et al. 2018a, b; Fetta et al. 2017; Pertíñez and Linares 2015; Velikonja et al. 2014).

Drill and practice exercises have been commonly conducted with word or list learning or paragraph recall tasks (Hart and Hayden 1986; Sohlberg and Mateer 1989; Tate 1997; Tsaousides and Gordon 2009). Gasparrini and Satz (1979) suggested that rote repetition with encouragement may be an effective treatment for memory problems. Although this condition was implemented as a control, participants showed significant improvement on a paired- associate memory task. However, it was noted that encouragement alone may result in improvements in performance, and this may have factored into the results. Moreover, a visual imagery mnemonic was still superior to the rote repetition with encouragement condition (Gasparrini and Satz 1979). In a study by Berg et al. (1991), memory-impaired patients completed either strategy training with multiple cognitive strategies, pseudo-rehabilitation (drill and practice of memory tasks and games), or received no training. On subjective measures, participants in both the strategy training and pseudo-rehabilitation groups reported significant improvements in memory capacity, insight into memory problems, coping with daily memory problems, and decreased anxiety about memory functioning. However, while the strategy training group showed significant improvements on objective memory performance measures after the training and at a later follow-up, the drill and practice group showed no significant changes, with similar scores to those of the control group throughout (Berg et al. 1991). Similar outcomes with drill and practice from various studies have resulted in these exercises being used as a control to account for general training effects (Doornhein and de Haan 1998; Glasgow et al. 1977; Kaschel et al. 2002; Schacter and Glisky 1986; Wilson 1982).

Based on the limited efficacy and transfer of drill and practice exercises, the strategies of the restorative approach have shifted toward CACR, which includes software designed to improve cognitive functioning, brain training programs, and virtual reality programs (De Luca, et al. 2018a, b; Gamito et al. 2015; Kim et al. 2011; Wentink et al. 2016; Yip and Man 2013). These strategies differ from traditional drill and practice exercises because they can be programmed to match the needs of the individual, include adaptive intensity of training, provide instant feedback, and can incorporate stimuli that simulate real-world situations and interactions to better facilitate generalization (Fernandez et al. 2017; Lebowitz et al. 2012; Maggio et al. 2019). It is expected that due to the adaptive intensity of the training and feedback, neural plasticity processes below the neuronal level (synapses are changed or created based on experiences) and at the neuronal network level (networks are altered due to changes in synapses and new synaptic connections) will be altered in such a way that can be sustained over time to maintain improvements seen during the CACR (Berlucchi 2011; Caeyenberghs et al. 2018; De Luca, et al. 2018a, b; Fernandez et al. 2017; Kane and Ward 2021). This targeted rehabilitation would then presumably translate to neuroplastic adaptations that are sufficient to result in functional level changes in daily life (Fernandez et al. 2017).

In a study by Fernandez et al. (2017), ABI patients were either allocated to an experimental group where they received CACR with RehaCom software or a control group who received standard rehabilitation over an eight-week period. The experimental group engaged with the software that provided compensatory strategies and training procedures to improve cognitive functions including memory and attention. In addition, there were individualized subprograms and levels of difficulty and participants received immediate feedback. The control group received similar training with activities targeted to improve cognitive functions including memory and attention using paper-and-pencil tasks. Both the experimental and control groups showed significant improvements across a variety of neuropsychological tests including memory tests. However, the experimental group showed significantly higher improvements in attention and memory following treatment compared to the control group. Thus, the authors suggest the superiority of CACR compared to a non-computerized approach, perhaps due to the incorporation of immediate feedback to improve self-awareness, higher levels of stimulation through improved stimuli quality and presentation to improve attention and focus, and dynamic adaptations to individual needs that increase motivation (Fernandez et al. 2017). Similar studies have highlighted improvements on cognitive tests when using CACR dedicated software such as RehaCom and CogMed in comparison with non-computerized approaches for individuals with ABI (De Luca, et al. 2018a, b; Lebowitz et al. 2012; Ressner et al. 2018; Yoo et al. 2015). However, limited effect on activities of daily living, small sample sizes, limited or no follow-up, and outcome measures that may not reflect ecologically relevant tasks demonstrate that there should be some caution when considering the effect of this type of CACR on functional outcomes and transfer of gains to real-world settings (De Luca, et al. 2018a, b; Fernandez et al. 2017; Ressner et al. 2018; Yoo et al. 2015).

The results with brain training programs such as Lumosity have been less promising, especially regarding generalization effects (Connor and Standen 2012; Simons et al. 2016; Wentink et al. 2016; Withiel et al. 2019). Wentink et al. (2016) compared a CACR intervention using Lumosity to a control condition involving education about the brain and stroke with stroke survivors across and eight-week period. They found limited effects of training for the intervention group compared to the control group and only for cognitive tests that were like the games in the training. In addition, no significant differences were found on subjective measures of cognition, self-efficacy, or quality of life. Thus, the authors suggest that targeting one cognitive domain may be more effective compared to targeting multiple domains and emphasize the need to tailor CACR training to individual needs of the participants (Wentink et al. 2016). Similar results with brain training programs have resulted in the suggestion that the effectiveness of these programs for use with individuals with ABI warrants further examination (Connor and Standen 2012; Wentink et al. 2016; Withiel et al. 2019). The contrast in support for memory rehabilitation programs such as RehaCom to the mixed outcomes with brain training programs such as Lumosity is worth noting. Although these approaches share similarities, it is possible that the divergence in evidence supporting the rehabilitation programs over the brain training programs relates to the target groups in development of the programs. For example, rehabilitation-specific programs may include aspects that are tailored to individuals suffering deficits in cognition, whereas brain training programs designed for improving cognition across a wide audience may not have the necessary adaptations required for individuals with cognitive deficits (Connor and Standen 2012; Simons et al. 2016; Withiel et al. 2019). Thus, it is perhaps essential to consider the target group for memory rehabilitation in relation to the features available within CACR programs.

Another strategy of CACR is virtual reality programs with both immersive and non-immersive options where virtual environments are created that allow users to engage with stimuli within that environment (Faria et al. 2020; Gamito et al. 2015; Kim et al. 2011; Maggio et al. 2019). Recent studies have demonstrated efficacy on improving task-related outcomes for individuals with ABI, posing virtual reality as a promising avenue in cognitive rehabilitation (Faria et al. 2020; Gamito et al. 2015; Kim et al. 2011; Maggio et al. 2019; Wilson 2013; Yip and Man 2013). However, current consensus indicates that further research is needed regarding the generalization to tasks in real life, training duration, and underlying mechanisms of virtual reality (Faria et al. 2020; Maggio et al. 2019; Velikonja et al. 2014; Yip and Man 2013).

There are several benefits to CACR, including immediate and dynamic feedback to participants, progressive learning, and customization to individual needs (Faria et al. 2020; Fernandez et al. 2017; Gamito et al. 2015; Wentink et al. 2016; Yoo et al. 2015). Immediate and dynamic feedback allows for improved self-awareness of current functioning level and motivation to continue with treatment, and progressive learning and adaptation to individual needs will prevent frustration or boredom, potentially improving adherence to the program (Faria et al. 2020; Fernandez et al. 2017; Lebowitz et al. 2012; Pertíñez and Linares 2015; Withiel et al. 2020). Moreover, some CACR programs can be administered at home and are relatively low-cost options, which may improve accessibility, particularly for individuals who have limited mobility options (Withiel et al. 2019; Yip and Man 2013). Another benefit of CACR, especially with virtual reality, is improved ecological validity of tasks that can be programmed to simulate real-life situations, which is difficult to attain using traditional paper-and-pencil methods (Faria et al. 2020; Gamito et al. 2015; Kim et al. 2011; Maggio et al. 2019; Wilson 2013; Yip and Man 2013). While these benefits of CACR are promising, barriers to compliance include the risk of fatigue impeding continuation of use, hemiparesis making using the technology difficult, negative feedback resulting in frustration and negative self-evaluations, lack of awareness of deficits, and computer literacy level (Connor and Standen 2012; Lebowitz et al. 2012; Pertíñez and Linares 2015; Wentink et al. 2016; Withiel et al. 2020; Yip and Man 2013). Moreover, there is limited evidence of transfer to activities of daily living or similar tasks (van Heugten and Wilson 2021; Wentink et al. 2016; Yoo et al. 2015). Recent reviews of CACR emphasized the wide variation of participant etiologies, small sample sizes, duration of time since injury and age span across research studies, making it difficult to draw conclusions regarding the effectiveness of improvements in cognitive functioning, although there appears to be some benefits for working memory (Fetta et al. 2017; López and Antolí 2020). CACR is a promising avenue for memory rehabilitation after ABI, yet further examination is warranted to determine the patients who would be able to access such a treatment approach and the training duration required to produce lasting effects.

### Summary

Although it was expected that drill and practice would be efficacious, especially if used with computer training, because computers could serve as a useful tool for repeated presentation and testing of stimuli, this was not the case (Kapur et al. 2002, 2004; Sohlberg and Mateer 1989). Additionally, improvements for memory of learned information through repetition does not indicate improvement in general memory functioning (Hart and Hayden 1986; Schacter and Glisky 1986). Overall, there appears to be little evidence supporting the use of repetitive drill and practice exercises in restoring function (Cicerone et al. 2000; Kapur et al. 2002; Prigatano 1987; Schacter and Glisky 1986; Tate 1997; Wilson 2009). However, CACR shows more promise in improving outcomes with the ability for dynamic adaptation to suit individual needs, a variety of tasks available, feedback to improve self-awareness, and the potential to include ecologically valid stimuli to promote generalization (Faria et al. 2020; Wilson 2013). Some barriers to CACR may include fatigue, hemiparesis, computer literacy, awareness of deficits, and negative self-evaluations resulting from negative feedback (Pertíñez and Linares 2015; Withiel et al. 2020). Moreover, further research is needed to demonstrate transfer effects to tasks in everyday life and to examine the required training duration and potential long-term effects of CACR (Withiel et al. 2019, 2020). Although CACR provides some promise for the restoration approach with regards to improvements on objective memory measures, further examination of the effects on functional outcomes and underlying mechanisms is warranted.

## Specific knowledge acquisition approach

Based on the limited evidence of success with drill and practice, an alternative approach was proposed: the *acquisition of domain-specific knowledge*. This entails teaching knowledge that is relevant to the individual’s everyday life in specific domains (e.g., names of hospital staff or remembering when and how to complete household duties) without the expected improvement in general memory functioning (Glisky and Schacter 1988; Kapur et al. 2004; Pino 2015; Schacter and Glisky 1986). Strategies within this approach include the vanishing cues method, errorless learning, and spaced retrieval (Censori et al. 1996; Clare and Jones 2008; Evans et al. 2000; Haslam 2017; Schacter and Glisky 1986). These strategies are thought to draw on preserved implicit memory abilities (that do not require conscious processing) to acquire specific knowledge or skills with appropriate training, instead of explicit memory abilities (that require conscious processing) which have been shown convincingly to be disrupted for memory-impaired patients (Baddeley and Wilson 1994; Clare and Jones 2008; Glisky and Schacter 1988; Haslam et al. 2011; Hunkin and Parkin 1995; Kessels and de Haan 2003; Wilson et al. 1994; Wilson and Fish 2017).

### Method of vanishing cues

This is a technique whereby as much cue information as is needed to produce the correct response is provided and then gradually withdrawn over several learning trials (Cheyne 1966; Haslam 2017; Kapur et al. 2004; Kessels and de Haan 2003; Moffat 1992). For example, in verbal memory tasks, a complete word is presented on the first trial and letters of the word are gradually removed from right to left as participants provide the correct response on each subsequent trial (Clare and Jones 2008; Glisky et al. 1986; Riley and Venn 2015). Incorrect responses are corrected by adding a letter until the participant can produce the word or by presenting the complete word (Evans et al. 2000; Glisky et al. 1986; Hunkin and Parkin 1995; Riley and Venn 2015). It has been proposed that the vanishing cues method would result in better recall than the standard anticipation method, where the complete word is shown after each guess regardless of response (Cheyne 1966; Hunkin and Parkin 1995). This proposal is based on the assumption that the vanishing cues method has similar characteristics as priming and would more efficiently tap implicit memory processes that remain intact for memory-impaired patients (Cheyne 1966; Glisky et al. 1986).

Glisky et al. (1986) assessed the vanishing cues method compared to a standard anticipation method. In the vanishing cues condition, memory-impaired patients were presented with definitions of computer-related words along with a fragment of the word, which contained one letter fewer than the participant required to correctly produce the word in the previous trial. In the standard anticipation condition, participants were shown the definition and guessed the word, followed by the presentation of the correct answer regardless of correct/incorrect guess. Across an eight-week period, the proportions of words correctly recalled across both conditions increased but with better performance in the vanishing cues condition (Glisky et al. 1986). Hunkin and Parkin (1995) were unable to replicate these findings and instead found a general improvement with both the vanishing cues and standard anticipation methods. The authors noted that a possible reason for the discrepancy is that their memory task required explicit memory and was therefore incompatible with the vanishing cues method (Hunkin and Parkin 1995). Recent evidence suggests that the type of instructions provided at the encoding stage during a vanishing cues tasks may influence outcomes (Riley and Venn 2015). Intentional instructions (i.e., those that encourage the use of explicit memory) can allow for more elaborate encoding during the task and can be used with individuals who have less severe memory deficits and are still able to capitalize on explicit memory abilities. In contrast, those who have more severe deficits may have more success with recall when provided with automatic instructions (i.e., those that encourage the use of implicit memory) during a vanishing cues task (Riley and Venn 2015). Thus, it is perhaps essential that the memory task and type of instructions provided align with the targeted memory ability.

The initial motivation for using the method of vanishing cues in rehabilitation was to provide information about computer terms required to use computers as external aids, but the technique has also been used with some success in learning face-name associations and list learning (Glisky et al. 1986; Glisky and Schacter 1988; Riley et al. 2004; Riley and Venn 2015; Thoene and Glisky 1995). However, it has been observed that the method of vanishing cues can be time-consuming because it may require many learning trials (Hunkin and Parkin 1995; Kessels and de Haan 2003; Riley et al. 2004). In particular, Glisky and Schacter (1988) noted that memory-impaired patients require many more learning trials than do controls. Moreover, on a transfer task where the wording of the vocabulary definition was altered (e.g., *loop* is changed from “a repeated portion of a program” to “if you want a program to perform the same operations repeatedly, you must put it in a ____”), control participants produced significantly more words without requiring letter cues than did the memory-impaired patients (Glisky et al. 1986). Based on these results and the difficulty for memory-impaired patients to respond to open-ended questions regarding the learned information, Glisky and Schacter (1988) noted that there are likely to be difficulties in transferring learned material between the laboratory and the real world when using the vanishing cues method with memory-impaired patients. Similarly, Riley and Venn (2015) suggest that the arbitrary list of words with limited practical relevance used in their study may have contributed to the attrition rate and suggest the use of more engaging and ecologically relevant stimuli in future research with the vanishing cues method.

### Errorless learning

This strategy involves preventing errors as much as possible during learning. Errorless learning is based on the assumption that memory for learned information will improve by reducing guessing and thus incorrect responses (Baddeley and Wilson 1994; Clare and Jones 2008; Shigaki et al. 2014; Wilson 2002, 2013). Errorless learning paradigms often include an errorful condition, where participants are provided with word stems (e.g., “BR”) and guess the word, and an errorless condition, where participants are provided with the correct word (e.g., “BREAD”) immediately after the stem (Baddeley and Wilson 1994; Evans et al. 2000; Fish et al. 2015; Wilson et al. 1994). It has been proposed that the errorful condition will introduce incorrect responses to the question through guessing, whereas the errorless condition eliminates that possibility, reinforcing only the memory trace for the correct information (Baddeley and Wilson 1994; Fish et al. 2015; Haslam et al. 2011; Wilson et al. 1994; Wilson and Fish 2017). Thus, this method may benefit memory-impaired patients who have difficulty correcting errors, which relies on explicit memory processes (Baddeley and Wilson 1994; Evans et al. 2000; Haslam et al. 2011; Kessels and de Haan 2003; Wilson and Fish 2017).

Baddeley and Wilson (1994) compared errorless and errorful learning across people with severe memory impairments, healthy older adults, and healthy young adults. Participants completed multiple learning and test trials in both errorless and errorful conditions. All three participant groups showed marked improvements when using the errorless learning method compared to the errorful method, but memory-impaired patients were significantly more affected by the advantage of the errorless learning compared to healthy young and older adults. This outcome is consistent with the view that memory-impaired patients have difficulty eliminating errors on their own, which can be resolved by training them with only correct information and reducing the possibility of encoding incorrect information through guessing (Baddeley and Wilson 1994). Comparable improvements using the errorless learning strategy with memory-impaired patients have been reported for learning person and object names, word lists, and face-name associations (Evans et al. 2000; Haslam et al. 2011; Wilson et al. 1994). In addition, it has been shown that errorless learning is superior to errorful learning for event-based prospective memory tasks for memory-impaired individuals, indicating a potential avenue for future research to explore the possibility of integrating errorless learning into activities of daily living (Fish et al. 2015; Wilson and Fish 2017). Similar to the method of vanishing cues, learning one item at a time over multiple trials may result in lengthy sessions for errorless learning (Evans et al. 2000; Wilson et al. 1994). Finally, the difficulty associated with achieving a truly errorless learning procedure has been noted as there are likely fewer rather than no errors compared to the errorful learning conditions (Clare and Jones 2008; Fish et al. 2015; Wilson and Fish 2017). As such, it has been suggested that errorless learning might be more aptly described as “error-reducing.”

### Spaced retrieval

Here, to-be-remembered information is presented and tested at increasing intervals, based on evidence that distributed practice results in better memory for information compared to massed practice (Censori et al. 1996; Creighton et al. 2013; Harris 1992; Haslam 2017; Wilson 2002). Mistakes are corrected immediately and if the information cannot be recalled, it is provided, reducing errors during learning (Clare and Jones 2008; Creighton et al. 2013). Additionally, when errors occur, the retrieval interval is reduced to the previous one before being progressively increased again (Haslam et al. 2011).

In a study by Schacter et al. (1985), memory-impaired patients completed spaced retrieval training over eight weeks using faces and experimenter-provided characteristics of the faces (names, occupations, hobbies). Participants showed improved recall of characteristic information from baseline to post-training assessment, which extended to those with severe memory deficits (Schacter et al. 1985). In addition, retrieval practice has been shown to have greater benefits for ABI patients in word and name recall in comparison with spaced restudy and massed practice (Evans et al. 2020; Sumowski et al. 2014). Although it has been implemented with ABI patients, spaced retrieval training is more often directed toward older adults with cognitive impairments or dementia patients and has been successful for these groups (Censori et al. 1996; Creighton et al. 2013; Evans et al. 2020; Haslam 2017; Haslam et al. 2011). While spaced retrieval has shown success with patients in experimental settings, previous research has been criticized for the low ecological validity of the stimuli used in the studies and the role of errorless learning as part of the technique remains unclear (Creighton et al. 2013; Haslam et al. 2011).

### Summary

Errorless learning, vanishing cues, and spaced retrieval strategies have shown positive outcomes in studies with memory-impaired patients. There is an advantage for errorless learning over errorful learning (Baddeley and Wilson 1994; Evans et al. 2000), for vanishing cues over standard anticipation (Glisky et al. 1986), and for spaced retrieval over uniform retrieval (Censori et al. 1996). These strategies have been designed with the intention to capitalize on preserved implicit memory abilities of memory-impaired patients (Censori et al. 1996; Haslam 2017; Kapur et al. 2004; Schacter and Glisky 1986; Wilson and Fish 2017). Therefore, it has been suggested that these techniques could be particularly beneficial for patients with severe memory impairments, who may have marked difficulties with tasks that require explicit memory (Censori et al. 1996; Clare and Jones 2008; Shigaki et al. 2014; Wilson 2002; Wilson and Fish 2017). Moreover, as these strategies include some error reduction at the learning stage, they may strengthen connections for only the correct stimulus and response associations for patients with poor error-monitoring (Clare and Jones 2008; Piras et al. 2011). However, these strategies require intensive training which can be time- and resource-consuming, requiring stimuli to be learned one at a time over multiple trials, and memory-impaired patients have required significantly more learning trials than healthy controls (Glisky and Schacter 1988; Kapur et al. 2002; Tailby and Haslam 2003). Finally, their generalizability has come into question given that the learned material is specific (e.g., word lists) that may not be relevant to everyday life needs (Creighton et al. 2013; Fish et al. 2015; Piras et al. 2011; Riley and Venn 2015; Tailby and Haslam 2003). However, to address this limitation, it has been suggested that patients may be able to quiz themselves or be quizzed by family members on learned information (e.g., phone numbers or names) or tested through the use of booklets, timers, or sliders (Evans et al. 2020; Fish et al. 2015; Moffat 1992; Sumowski et al. 2014). Further exploration of these strategies focusing on their impact and implementation in everyday life is warranted.

## Compensatory approach

The compensatory approach involves using strategies and tools that alleviate the impact of memory problems in daily life and improve daily functioning without the expectation of improvement of memory functioning (Cicerone et al. 2000, 2005; Schacter and Glisky 1986; van Heugten and Wilson 2021). A compensatory approach may become the focus of memory rehabilitation when restoration of function seems to be no longer possible either through spontaneous recovery or memory training (De Luca, et al. 2018a, b; Wilson 2000). It is held that the compensatory approach can lead to improvements as a result of a structural reorganization of neural modules (das Nair and Lincoln 2012; Rothi and Horner 1983; Shigaki et al. 2014). Specifically, training patients to use compensatory strategies may result in the reorganization of neural networks to recruit undamaged circuits to compensate for the lack of activity in the damaged circuits (das Nair and Lincoln 2012; Galetto and Sacco 2017; Rothi and Horner 1983; Tate 1997). Compensatory strategies include internal and external memory aids (das Nair et al. 2016; Intons-Peterson and Fournier 1986; Wilson 2000).

### Internal aids

These involve the mental manipulation of information via imagery or associations to improve retrieval at a later time (das Nair and Lincoln 2012; Haskins et al. 2012; Lewinsohn et al. 1977; O’Neil-Pirozzi et al. 2010, 2016; Perna and Perkey 2016; Wilson 1982). Internal aids include mnemonics such as chunking, rhyming, acronyms, visualization, first letter mnemonics, and chaining (Crovitz 1979; das Nair et al. 2016; Harris 1992; Lewinsohn et al. 1977; Velikonja et al. 2014). Specific strategies include the method of loci, the peg method, the face-name mnemonic technique, and the story method (Intons-Peterson and Fournier 1986; Madan 2014; Tate 1997). Across internal aids, mnemonic strategies with imagery components are the most commonly evaluated and show the most promising results (Censori et al. 1996; O’Neil-Pirozzi et al. 2016; Patten 1972; Pino 2015). Internal aids are often used as part of memory rehabilitation or training rather than as standalone memory aids (das Nair and Lincoln 2012; Leśniak et al. 2018; Lewinsohn et al. 1977; O’Neil-Pirozzi et al. 2010; Perna and Perkey 2016; Velikonja et al. 2014).

In a study by Kaschel et al. (2002), patients with mild memory impairment were taught to rapidly generate images when provided with verbal information that was either autobiographical (e.g., remember a holiday) or neutral (e.g., change a lightbulb). They were then shown videos of objects or actions and interacted with them to varying difficulty levels (ranging from holding the image in their mind to drawing the image). In comparison with a pragmatic group who received a variety of internal and external memory aid training, the imagery group showed significant improvements on immediate and delayed story recall, and an improvement in relatives’ rating of everyday relevant changes (Kaschel et al. 2002). More recently, the self-imagination method, a strategy which involves visual imagery and semantic elaboration, has been developed to capitalize on preserved mnemonic mechanisms related to the self in memory-impaired individuals (Grilli and Glisky 2010, 2011). Across two studies testing the mnemonic, memory-impaired participants demonstrated improvements in recognition memory and delayed cued recall (Grilli and Glisky 2010, 2011). Additionally, these studies demonstrated an advantage for this mnemonic over others such as visual imagery, semantic elaboration alone. (Grilli and Glisky 2010, 2011). These findings were then extended to demonstrate the benefits of the self-imagination effect for a prospective memory task for memory-impaired individuals, highlighting a potential avenue for future research investigating the application of this mnemonic strategy within real-life settings (Grilli and McFarland 2011). Similarly, other researchers have demonstrated improvements in memory for memory-impaired patients using chaining, the peg method, the face-name mnemonic technique, and first letter mnemonics (Crovitz 1979; Glasgow et al. 1977; Lewinsohn et al. 1977; Patten 1972; Wilson 1982).

Proposed criteria for the success of mnemonics in improving daily functioning are that maintenance of the strategy by the individual must be present exclusive of explicit instructions after initial learning and the strategy must be generalizable to other tasks (Leśniak et al. 2018; Schacter and Glisky 1986). It has been suggested that internal memory aids are effective because they require a deeper level of processing, encourage integration between separate pieces of information, and include internal retrieval cues (Leśniak et al. 2018; O’Neil-Pirozzi et al. 2016). There is some evidence for the effectiveness of using internal memory aids in experimental settings, but evidence regarding the generalizability to untrained tasks that affect functioning in everyday life is more limited (Censori et al. 1996; O’Neil-Pirozzi et al. 2016; Tate 1997). It is important to note that internal strategies may only be beneficial for patients with mild to moderate memory impairments because they require manipulation of information (Haskins et al. 2012; Schacter and Glisky 1986; Shigaki et al. 2014). In addition, internal aids require a certain level of self-awareness regarding memory deficits in addition to self-monitoring and self-regulation to make continued efforts to apply learned strategies in appropriate situations (Velikonja et al. 2014). Finally, examination of the potential long-term effects of internal memory strategies is warranted (O’Neil-Pirozzi et al. 2010, 2016).

### External aids

These involve manipulation of the environment or the use of devices to store information that can be accessed at a later time, reducing memory demands (Harris 1980; Intons-Peterson and Fournier 1986; Wilson 2000). Environmental adaptations include labeling doors or items in the house, using arrows for directions, and strategically placing objects in specific locations relevant to when or how they are to be used (Evans et al. 2003; Jamieson et al. 2017; Kapur et al. 2002; Tate 1997). Memory aids include notebooks, calendars, personal digital assistants, and mobile phones that can be used for shopping lists or reminders for appointments or to take medications (Evans et al. 2003; Harris 1992; Kapur et al. 2004; Schacter and Glisky 1986; Velikonja et al. 2014; Wilson 2002). External aids may benefit individuals who have difficulty learning or using the previously outlined internal strategies (Kapur et al. 2002).

Sohlberg and Mateer (1989) proposed a structured, systematic approach to training memory-impaired individuals on using a memory notebook to record orientation, daily activities, appointments, transportation information, names, and feelings. In a case study, a 19-year-old patient with severe memory deficits learned the sections of the notebook in an acquisition phase, learned how to record in the book through an application phase, and finally used the notebook in naturalistic settings in an adaptation phase. Following six months of intensive training, the patient was able to independently use the notebook system. Although his profound memory and learning difficulties persisted, he required minimal daily assistance in everyday tasks and increased his production at work. The authors suggested that similar systematic training should be employed with other external aids as patients must understand how and when to use them for them to be effective (Sohlberg and Mateer 1989).

Advances in technology have allowed for further examination of systems such as NeuroPage, prosthetics, and smart house options to be used as external aids (Boman et al. 2010; Hersh and Treadgold 1994; Jamieson et al. 2017; Vasquez et al. 2021; Wilson et al. 1997). NeuroPage is a portable paging system for reminders and cues that can be personalized to each individual and circumvent the issue of forgetting to use the external memory aids such as notebooks and has demonstrated success with memory-impaired individuals (Jamieson et al. 2017; Wilson et al. 1997). However, the use of pagers in general as assistive technologies has decreased as much of their functions are now included within the capacity of mobile phones (Jamieson et al. 2017; Lannin et al. 2014; Wong et al. 2017). Since the introduction of NeuroPage, researchers have further explored digital prosthetic aids with promising generalization and long-term effects (Ferguson et al. 2015; Svoboda et al. 2012, 2015; Vasquez et al. 2021). Boman et al. (2010) examined the use of a home-based electronic memory aid based on sensors that indicate whether an activity was already completed and then provides reminders as needed. The product was found to be helpful for four out of five of the participants and highlights the possibility of using smart house technology to help in the compensation of daily functioning for ABI survivors (Boman et al. 2010). Lannin et al. (2014) demonstrated the advantages of using personal digital assistants to reduce memory failures compared to non-electric compensatory aids whereas Svoboda et al. (2012, 2015) successfully trained moderate and severe memory-impaired individuals to use personal digital assistants and mobile phones to improve daily functioning outcomes that were sustained over a long-term period. These studies have demonstrated the evolution and beneficial effects of assistive technologies that are more versatile and portable than older external aids such as notebooks and calendars (Boman et al. 2010; Jamieson et al. 2017; Lannin et al. 2014; Svoboda et al. 2012, 2015; Vasquez et al. 2021; Wong et al. 2017). However, there remains a need for extensive training to adapt usage of these devices into prosthetic aids, which must be considered when prescribing the use of external memory aids (Svoboda et al. 2015; Vasquez et al. 2021; Velikonja et al. 2014; Wong et al. 2017).

External memory aids are the dominant choice amongst persons with ABI and healthy adults (Evans et al. 2003; Harris 1980; Intons-Peterson and Fournier 1986). Presumably, external compensatory strategies are favored in comparison with internal strategies or knowledge acquisition strategies for practical reasons because people are already familiar with them, it is perhaps simpler and quicker to train people to use them, and they may be easier to use in a variety of situations (Chouliara and Lincoln 2016; Zencius et al. 1990, 1991). One survey revealed that the four most commonly used external memory aids were calendars, wall charts, notebooks, and lists (Evans et al. 2003). However, with the rise in prevalence of technology, the use of aids such as mobile phones and alarms/timers has increased (Jamieson et al. 2017; Wong et al. 2017). It has been suggested that the benefit of external memory aids comes from increased functional ability and self-confidence in patients who use them (Ferguson et al. 2015; Kapur et al. 2004). External aids have a particularly difficult limitation: patients must be aware of their memory limitations in order to be motivated to use them (Pino 2015; Shigaki et al. 2014). However, it has been acknowledged that the need to remember to use external aids may be reduced with the use of electronic aids which can be programmed to provide reminders or alarms (Jamieson et al. 2017; Lannin et al. 2014). It is important to note that the effectiveness of external aids might depend on several factors including the device, software changes that may alter the interface of technological aids, the age of the patient, the time since onset of memory impairment, and the extent to which the aids were used before impairment (Censori et al. 1996; Jamieson et al. 2017; Piras et al. 2011; Svoboda et al. 2012). Finally, although external memory aids are recommended to patients or used as part of rehabilitation programs, there is little information available as to the extent of the training required to use external aids efficiently in daily life as this may vary by each individual’s needs (Haskins et al. 2012; Sohlberg 2005; Velikonja et al. 2014; T. K. Wade and Troy 2001).

### Summary

There has been some success in applying internal and external memory aids to improve the daily functioning of memory-impaired individuals. It has been suggested that internal aids would be better suited to individuals with mild memory impairment, whereas external memory aids may be better suited to individuals with moderate to severe impairment (Cicerone et al. 2011, 2019). However, while patients may successfully apply these strategies when provided with instructions during rehabilitation sessions, they may face more difficulties when applying strategies independently (Censori et al. 1996; Shigaki et al. 2014; Wilson 2002). Therefore, careful consideration should be given to the strategies that best suit the needs of the memory-impaired patient.

## Holistic approach

The holistic approach addresses the emotional, social, and functional consequences of ABI alongside the cognitive consequences (Ben-Yishay 2000; Prigatano 2013; van Heugten and Wilson 2021; Wilson 2013). Key factors in this approach involve (1) establishment of a therapeutic setting or community, (2) addressing knowledge of the deficit and subsequent impact on functional abilities through education, (3) addressing the cognitive deficit, usually with compensatory aids, (4) addressing the emotional and social consequences of the deficit through psychotherapy, and (5) involving family members in the rehabilitation process (Ben-Yishay 2000; Nilsson et al. 2011; Prigatano 2013; Withiel et al. 2019). This approach is underpinned by the notion that the multidimensional nature of cognition and the interaction with the other systems should be addressed together because they interact with and influence each other (Afsar et al. 2021; Ben-Yishay 2000; Perna and Harik 2020; Prigatano 2013; Wilson 2013). The ultimate goal is to address the consequences of ABI in a manner that will allow for establishment of satisfactory life through community integration, social participation, and productivity whilst managing continued limitations (Cicerone et al. 2008; Exner et al. 2021; Holleman et al. 2018; Shany-Ur et al. 2020).

The holistic approach has been examined in comparison with a standard multidisciplinary approach for individuals with TBI by Cicerone et al. (2008). In this study, half of the participants were allocated to the standard neurorehabilitation program where they received interventions targeting specific deficit areas such as cognitive functions and were primarily conducted with individual therapies with some limited options for group treatments available. The other half of the participants were allocated to the intensive cognitive rehabilitation program which were conducted in group sessions consisting of therapist and peer feedback, compensatory strategies, and an emphasis on metacognition. While both groups showed significant improvements on neuropsychological functioning measures, the holistic program resulted in a moderate positive effect on community functioning and small effect on life satisfaction, neither of which were observed with the standard program. The authors suggest that metacognition and emotional regulation aspects of the holistic program played an important role in the difference between the two programs. The results of this study highlight the beneficial effects of supporting the psychosocial, emotional, and metacognitive aspects of ABI alongside the cognitive consequences, however, it is also mentioned that further examination of the contributions of individual components of the program is warranted (Cicerone et al. 2008).

In a study by Shany-Ur et al. (2020), ABI patients in community settings participated in a holistic program over the course of 10 months and then completed follow-up assessments at one-, two-, and three-year post-completion. Cognitive domains addressed included memory, attention, and executive functioning. Psychological interventions included individual and group therapy, and family members were involved in psychoeducational group meetings and optional family therapy. Significant improvements were found in employment rates following the program and work stability during the follow-up period. Additionally, community integration and perceived quality of life significantly improved across time. However, there was no significant difference between time points on mood disturbance, and the authors suggest that perhaps increased self-awareness of deficits because of the program resulted in lowered mood. Moreover, it was highlighted that the length of the treatment program was considerable, which may limit the generalization of the findings as rehabilitation length may differ by institution or country. Finally, most of the participants were unemployed at the time of enrolment but gained employment at a later period. This highlights an important interaction between program length and return to work, which may occur during the program for some individuals, potentially resulting in attrition. The results of this study demonstrated the beneficial and stable long-term effects of holistic neuropsychological rehabilitation but also highlighted some areas that require further consideration (Shany-Ur et al. 2020). Similar studies have found positive effects of holistic rehabilitation on psychological, emotional, and quality of life measures, although the benefits on cognitive functions have been mixed (Afsar et al. 2021; Exner et al. 2021; Holleman et al. 2018; Nilsson et al. 2011; Sarajuuri et al. 2018; Urech et al. 2020).

## Summary

The benefits of the holistic approach may not only lie in the potential improvements in daily functioning, but also community participation, productivity, self-efficacy and life satisfaction (Cicerone et al. 2019; Nilsson et al. 2011; Wilson 2013). Participant perspectives have revealed that knowledge and understanding of brain functions and the impact of ABI are beneficial aspects of holistic programs because they provide participants with information that reduces stress regarding current ability levels (Chouliara and Lincoln 2016; Nilsson et al. 2011). Thus, it appears that improving self-awareness through education and feedback within the therapeutic setting while addressing the emotional, social, and behavioral consequences of ABI can promote improved functional outcomes in these areas. Although the evidence regarding the improvement of cognitive functions is more mixed, re-integration within community settings and improvements in social participation are undeniably beneficial outcomes of the holistic approach. However, there are some limitations worth noting: first, it is difficult to identify the contributions of specific aspects of the program and thus the optimal combination of strategies to form a program, second, there are considerable time and resource commitments requiring cost considerations, and finally, motivation to participate by all parties involved (patient and family members) need to be aligned to fit within the model (Chouliara and Lincoln 2016; Cicerone et al. 2008; Exner et al. 2021; Prigatano 2013). Altogether, there is good evidence that the holistic approach can improve the lives of ABI patients, and it will likely be adapted more frequently in the future with some essential considerations regarding cost, delivery, and motivation (Nilsson et al. 2011; Prigatano 2013; Wilson 2013).

## Comparing approaches

Compensatory approaches are used more often than restorative approaches in memory training and rehabilitation but they are sometimes combined to form a multi-strategy program (Haskins et al. 2012; Lincoln et al. 2002; Raskin et al. 2019; Robertson 1999). It has been suggested that the preference for compensatory approaches is due to the lack of evidence supporting lasting improvement following restoration approaches (Evans et al. 2003; Robertson 1999). Similarly, limited support for the generalization of drill and practice has resulted in the exploration of the specific knowledge acquisition approach (Thoene and Glisky 1995). Finally, the emergence of the holistic approach takes into consideration emotional, social, and behavioral factors of ABI that are not commonly addressed across the other approaches (Prigatano 2013; Wilson 2013). A few studies have directly compared the different memory rehabilitation approaches.

Thoene and Glisky (1995) compared the specific knowledge acquisition approach using the vanishing cues strategy, the compensatory approach using a mnemonic strategy, and an exposure alone intervention with a video presentation on face-name recall for memory-impaired patients. The only condition in which all patients were able to reach the criterion (recalling four first and last names) was the mnemonic condition. Moreover, the participants needed significantly fewer trials to reach criterion with the mnemonic condition than with the vanishing cues or the video presentation conditions, which did not significantly differ from each other. The researchers proposed that the results of this study reflect the need to correctly match the stimulus type to the strategy type (Thoene and Glisky 1995). Specifically, the mnemonic strategy encouraged connecting the names and the faces, whereas the vanishing cues training emphasizes learning individual items such as the names but not the connection between the names and the faces. Moreover, the abstract nature of person names was manipulated to be less abstract in the mnemonics condition where semantic associations were used to connect the name and the face, but in the vanishing cues condition, the emphasis was on learning the text. In this instance, the authors proposed that the compensatory approach was superior to the specific-knowledge acquisition approach because it was more appropriately matched to the stimulus type. Similarly, Evans et al. (2000) found that the errorless learning method is beneficial for (first letter) cued recall of names, requiring implicit memory but not for arbitrary face-name associations with free recall, requiring explicit memory. However, a combination of an imagery strategy and errorless learning led to improvements in free recall of names (Evans et al. 2000). These studies have highlighted the importance of using the memory rehabilitation approach that is most compatible with the stimulus types.

das Nair and Lincoln (2012) compared the restorative and compensatory approaches with a self-help approach. Individuals who reported memory problems due to ABI engaged in training sessions with a researcher and completed homework exercises. The restorative and compensatory experimental groups learned about internal memory aids and errorless learning techniques. The compensation group was additionally taught how to employ external memory aids. The restoration group was taught how to encode information using “Who,” “What,” “Why,” “When,” “Where,” and “How” questions, attention retraining exercises, and letter and number cancellation tasks. The self-help group was taught relaxation and coping techniques but no memory strategies. The restoration and compensation groups had significantly higher scores on the Internal Memory Aids Questionnaire at five and seven-month follow-up points. However, there were no significant differences between any groups on the primary outcome measure, the Everyday Memory Questionnaire. In general, the restoration and compensation groups showed similar outcomes. The authors reported that the restoration group also developed the use of some compensatory strategies on their own, but without a baseline test of the External Memory Aids questionnaire, this can only be considered an observation by the researchers. Based on the few significant outcomes, limited sample size, and heterogeneity of the sample, the authors concluded that concrete assertions are not warranted regarding the effectiveness of the memory rehabilitation approaches in this study (das Nair and Lincoln 2012).

Withiel et al. (2019) compared the restorative approach using computer-assisted cognitive rehabilitation (CACR) to a more holistic approach involving memory skills group-based training and a waitlist control group. Community dwelling stroke survivors set memory-specific rehabilitation goals with a trained researcher before being randomly assigned to the CACR, memory skills, or control group. The CACR was conducted using Lumosity involving games targeting memory function and was completed by participants at their home with weekly phone calls with the researchers to assess compliance. The memory skills group consisted of education regarding memory, internal and external strategies, and lifestyle sessions facilitated by a neuropsychologist. There was no significant difference between the groups on individualized goal attainment at baseline. Participants in the memory skills group demonstrated significantly greater individualized goal attainment from baseline to post-intervention in comparison with the waitlist control. In addition, these gains were maintained at follow-up six weeks later where the memory skills group had significantly greater goal attainment compared to other two groups. However, the differences between the groups on objective measures of memory were more mixed with little group differences across visual and verbal learning and memory measures and limited differences on prospective measures. Subjective measures revealed a significant reduction in the frequency of everyday memory complaints for participants in the memory skills group from baseline to post-intervention but not follow-up, and the CACR group had a significant reduction in prospective memory failures post-intervention but not at follow-up. The results of this study highlight an advantage for the holistic approach in relation to waitlist control and CACR for functional improvements. However, several of the measures used in this study were subjective and may therefore be subject to biases. In addition, the memory skills group was in regular contact with other stroke survivors and the researchers, whereas the CACR and the control groups had fewer interactions, which may have influenced responses to goal attainment outcomes and subjective memory measures. The authors suggest that memory skills group provides a more holistic approach to memory rehabilitation which may better address the multifaceted nature of memory deficits following a stroke (Withiel et al. 2019). In a follow-up qualitative study involving the same participants, increased knowledge and understanding of memory functioning and strategies were reported in both groups; however, these were employed in different manners such that the CACR group developed strategies aimed at improving their experience with the training, whereas the memory skills groups developed strategies in a structured manner and then applied them to everyday situations (Withiel et al. 2020). Additionally, connecting with others is a theme that was mentioned by the memory skills but not the CACR group. This is a result of the circumstances in which the trainings were completed but highlighted the importance of social interaction, cohesion, and sharing of knowledge and experiences that may be particularly beneficial in a holistic approach (Withiel et al. 2020).

Rather than comparing approaches, some studies have examined the outcomes of *combining* different approaches or strategies within an approach. CACR has been found to have some positive effects on cognitive task outcomes when combined with traditional cognitive therapy (Ressner et al. 2018), brain stimulation (Yin et al. 2020), and virtual reality (Kim et al. 2011). It has been suggested that a combination of CACR and group-based memory training may offer the opportunity for social interaction, an important aspect often highlighted with group training, and the opportunity to address the training using technology (Leśniak et al. 2018; Withiel et al. 2019, 2020). A combination of specific knowledge acquisition and compensatory techniques was used by Svoboda et al. (2012, 2015) to successfully train memory-impaired individuals to use smartphones and personal digital assistants to improve daily functioning. In phase 1, core steps of the training included entering and saving an event in a calendar application of the device using errorless learning and vanishing cues whereby cuing support was provided when entering calendar events and gradually removed. In phase 2, new steps were added in subsequent sessions that would generalize to real-life situations and allow for continued use of the device post-intervention. The incorporation of errorless learning and vanishing cues was based on the theory that implicit memory is preserved following brain injury, so that learning of a skill remains possible, which is utilized to train usage of the technological device that can then be used as a compensatory aid. The results revealed that across the first ten training sessions, participants acquired the skills to use the calendar application with less and less support required. In addition, participants were able to use the devices in their daily lives to reduce memory mistakes, which was maintained at a long-term follow-up study (Svoboda et al. 2012, 2015; Vasquez et al. 2021). Raskin et al. (2019) found some improvements on training tasks, neuropsychological measures, and generalization measures for brain injury patients when examining a combination of visual imagery strategies under the compensatory approach and rote repetition under the restorative approach. However, there were no significant differences between post-treatment and a one-year follow-up on any of the measures, possibly suggesting that the dosage of the intervention and booster sessions may improve long-term outcomes (Raskin et al. 2019). Similar positive outcomes with combined approaches have resulted in the suggestion that an optimal approach to treating memory deficits following ABI may require a combination of approaches and strategies carefully designed to account for patient abilities and appropriately address individual needs (Lannin et al. 2014; López and Antolí 2020).

### Efficacy of memory rehabilitation interventions

There is little conclusive evidence indicating whether one type of rehabilitation approach is more effective for memory rehabilitation because the appropriate approach may vary based on a multitude of patient characteristics and availability of resources (Barman et al. 2016; Cicerone et al. 2019; das Nair and Lincoln 2012). However, there is a preference for compensatory strategies in rehabilitation and many programs contain a combination of internal and external memory aids (Censori et al. 1996; Doornhein and de Haan 1998; Haskins et al. 2012; Perna and Perkey 2016; Shigaki et al. 2014). In a case study by Wilson (1982), a program developed for a memory-impaired patient included visual imagery, the peg method, and first letter mnemonics. Ryan and Ruff (1988) implemented a memory retraining intervention involving chaining, rehearsal, and visual imagery in addition to external aids. Intervention programs for TBI patients have included internal memory strategies such as clustering, chaining, and imagery techniques as supplements to external memory aids that patients were already using (O’Neil-Pirozzi et al. 2010).

Systematic reviews of cognitive rehabilitation over the past two decades have consistently recommended a combined use of internal and external compensatory strategies as memory training for patients with mild to moderate memory impairments (Cicerone et al. 2000, 2005, 2011, 2019). Errorless learning and external aids such as electronic technologies have been recommended for persons with severe memory impairment (Cicerone et al. 2005, 2011, 2019). Holistic neurorehabilitation programs may provide short and long-term benefits in cognitive, function, and psychosocial outcomes which can improve independent living, societal participation, emotional well-being, and quality of life (Cicerone et al. 2011, 2019). A review of literature on cognitive interventions after TBI by Rees et al. (2007) concluded that there is strong evidence for the use of external memory aids to improve daily functioning but not the underlying memory deficits and limited exploration of the long-term possibility of using external memory aids. It was also concluded that there is strong evidence that internal memory aids are beneficial for TBI survivors with mild but not severe impairment, but little information remains about the sustained effect (Rees et al. 2007). Following a systematic review of cognitive rehabilitation studies with stroke patients, das Nair et al. (2016) concluded that although participants in memory rehabilitation groups report better memory outcomes on subjective assessments compared to those in the control groups, these results do not persist in the long term, nor does there appear to be a cohesive effect of memory rehabilitation on objective memory tests, functional abilities, or quality of life across studies. Participants in these studies likely believe their memory has improved as they learn more about memory and techniques they can use (das Nair et al. 2016). Thus, there is some indication that external memory aids and knowledge acquisition strategies may benefit patients with moderate to severe memory impairments, whereas internal memory aids are more suitable for those with mild memory impairments. Moreover, there is some indication that the holistic approach may promote increased participation, emotional well-being, and quality of life. Most importantly, it has been suggested that the effectiveness, long-term impacts, and interaction of rehabilitation with severity of memory impairment be further explored.

### Factors that influence memory rehabilitation outcomes

In addition to cognitive impairments following ABI, deficits in metacognition and emotional disturbances including depression and anxiety can be present and impact daily functioning and community participation such as returning to work (Afsar et al. 2021; Haskins et al. 2012; Long et al. 2014; Perna and Harik 2020; Robertson and Schmitter-Edgecombe 2015). Metacognition refers to the knowledge of one’s own cognitive abilities and includes self-awareness, self-monitoring, and self-regulation (Al Banna et al. 2015; Robertson and Schmitter-Edgecombe 2015; Sansonetti et al. 2021). Self-awareness is the perception and cognitive reaction to one’s own abilities across different domains including cognitive, physical, social, and psychological (Long et al. 2014; Sansonetti et al. 2021). Deficits in self-awareness are correlated with cognitive deficits and an individual’s level of self-awareness regarding the nature and severity of memory impairment can influence participation in daily activities such as return to work and also in cognitive rehabilitation (Al Banna et al. 2015; Berlucchi 2011; Lexell et al. 2013; Long et al. 2014; Perna and Harik 2020; Robertson and Schmitter-Edgecombe 2015). For example, individuals who are unaware of their memory deficits may not seek help, whereas those who are aware of their deficits may elect to be involved in rehabilitation programs or studies, resulting in biased samples. In a different capacity, receiving feedback and communication with others in a group setting as part of rehabilitation have been deemed factors that positively influence self-awareness (Chouliara and Lincoln 2016; Cicerone et al. 2008; Long et al. 2014; Nilsson et al. 2011).

Higher levels of metacognitive skills such as self-awareness are correlated with better task and functional outcomes, whereas impaired self-awareness is linked to poorer functional outcomes (Al Banna et al. 2015; Sansonetti et al. 2021). In addition, increased awareness of deficits due to education, social support, and feedback can provide opportunities for acceptance and understanding of the adaptations that are needed in daily life (Long et al. 2014; Nilsson et al. 2011; Sansonetti et al. 2021). However, it is important to note that the process of self-awareness through education and psychotherapy may require gradual changes and different amounts of time for different individuals (Nilsson et al. 2011; Sansonetti et al. 2021). Although self-awareness is a common component of holistic programs and infrequently an aspect of the compensatory approach, it is essential to note that some level of self-awareness is required to engage with any type of rehabilitation (Leśniak et al. 2018; Lexell et al. 2013; Urech et al. 2020; Velikonja et al. 2014). Although it has been suggested that self-awareness should be measured and addressed, there is some debate regarding the efficacy of the current measures of self-awareness and treatment interventions (Al Banna et al. 2015; Long et al. 2014; Sansonetti et al. 2021). Given that self-awareness is frequently impacted following ABI and can negatively impact functional outcomes, it should be considered an important aspect of each rehabilitation approach.

Emotional disturbances such as anxiety and depression can be prevalent and persist following ABI (Gould et al. 2014; Perna and Harik 2020; Slenders et al. 2020; Terry et al. 2019; van Heugten and Wilson 2021). Previous research has demonstrated correlations between cognitive impairments and anxiety and depression following ABI (Gould et al. 2014; Li et al. 2021; Spitz et al. 2013; Terry et al. 2019). Emotional disturbances can negatively influence social integration, return to work, and quality of life (Exner et al. 2021; Gould et al. 2014; Slenders et al. 2020). Factors such as worry, anxiety, and depression may negatively influence initial decisions to participate in memory rehabilitation. It has been found that attrition from rehabilitation studies are more likely to occur with more depressed participants and anxiety can increase as a result of finding out about one’s current level of progress (Chouliara and Lincoln 2016; Miller and Radford 2014; Svoboda et al. 2015; Withiel et al. 2020).

Alternatively, rehabilitation may positively influence emotional adjustment to the impact of memory impairment (Chouliara and Lincoln 2016). For example, within a group-based setting, embarrassment may be reduced upon recognition that other individuals share similar forgetting experiences, or negative self-evaluations may be diminished upon learning about the impact of ABI on cognition (Chouliara and Lincoln 2016; Lexell et al. 2013; Withiel et al. 2020). Stress and anxiety management are likely important aspects of rehabilitation programs, and while they are incorporated within the holistic approach, they should be considered when applying any rehabilitation approach (Lexell et al. 2013; Urech et al. 2020). Although pharmacological interventions may be used to treat depression and anxiety, these issues may require further consideration (Perna and Harik 2020; Urech et al. 2020; van Heugten and Wilson 2021). Altogether, it has been suggested that incorporating several factors such as metacognition and emotional consequences of ABI into cognitive rehabilitation will improve functional outcomes (Al Banna et al. 2015; Nilsson et al. 2011).

## Limitations of current approaches

There are several general limitations to current memory rehabilitation approaches. Early restorative strategies such as drill and practice are relatively ineffective, whereas CACR has limited generalization effects at the current stage (López and Antolí 2020). Specific-knowledge acquisition strategies can be time- and resource-consuming as they require multiple learning trials, and the ecological validity of the stimuli used in these strategies is relatively low (Schacter and Glisky 1986; Wilson et al. 1994). Internal compensatory aids are often taught in controlled settings with selected stimuli, resulting in little change to daily functioning because of the difficulty of applying it in different contexts with novel stimuli (Censori et al. 1996; Kapur et al. 2002). External memory aids require patients to be aware of their deficit and remember to use the tools, which is a memory task in itself, and may require intensive training (Kapur et al. 2004; Wilson 2000). Finally, the holistic approach attempts to address multiple consequences of ABI, but the optimal combination of strategies is unclear, and the length of the programs may result in considerable time and resource requirements (Prigatano 2013; Shany-Ur et al. 2020).

There is a wide variety in the structure of memory rehabilitation programs. There is no consensus for the treatment method, frequency, or duration of treatment with some ranging from two weeks to several months and each session lasting between 30 min and two hours (das Nair et al. 2016; Dou et al. 2006; Kaschel et al. 2002; López and Antolí 2020; O’Neil-Pirozzi et al. 2010, 2016; Piras et al. 2011). Some programs provide individual sessions, whereas others involve group rehabilitation, which may result in different therapeutic effects (das Nair et al. 2016; Leśniak et al. 2018; O’Brien et al. 2013; O’Neil-Pirozzi et al. 2010; Zucchella et al. 2014). Across cognitive rehabilitation studies, there is often a mix of etiologies including TBI, stroke, and multiple sclerosis, making it difficult to draw conclusions about efficacy of rehabilitation for specific populations (das Nair and Lincoln 2012; Fetta et al. 2017; Haslam et al. 2011; Perna and Perkey 2016; Riley and Venn 2015; Wilson et al. 1994). Similarly, severity of memory impairment has either been not reported (Doornhein and de Haan 1998; Dou et al. 2006; Gasparrini and Satz 1979) or assessed with different measures, which limits the ability to assess the efficacy of strategies for across different severity levels (Evans et al. 2000; Kaschel et al. 2002; O’Neil-Pirozzi et al. 2010; Piras et al. 2011; Riley and Venn 2015; Ryan and Ruff 1988; Tailby and Haslam 2003). Additionally, subjective memory or cognitive complaints are an inclusion criterion in some studies, indicating that these studies may be biased toward individuals who are aware of their memory deficits and motivated to seek help (Nilsson et al. 2011; Wentink et al. 2016; Withiel et al. 2019). Finally, self-awareness of memory deficits along with emotional disturbances and impact on daily functioning is infrequently addressed across studies, with few studies using screening measures for self-awareness and those that include a measure having unclear definitions (Al Banna et al. 2015; Jamieson et al. 2017; Sansonetti et al. 2021; Svoboda et al. 2015). Therefore, it is difficult to compare the effect of rehabilitation programs or strategies across studies due to the variability in etiologies, assessment methods, classification of memory impairment severity, and different paradigms.

Across some studies, the differences between the three approaches have been confounded. Schacter and Glisky (1986) stated that restoration of memory function has been attempted by “repetitive drill or practice that aims to ‘strengthen’ damaged memory processes, or training in special memory strategies to compensate for defective processes” (p. 259). Here, both drill and practice and mnemonics were considered under the restoration approach. das Nair and Lincoln (2012) indicated that “restitution is attempted by drill and practice on focused, discrete aspects of a cognitive function” (p. 1). However, “participants in both memory intervention programs were taught the use of internal memory aids and errorless learning techniques” (das Nair and Lincoln 2012, p. 2). Thus, the restitution program included internal compensatory strategies and specific knowledge acquisition strategies. O’Neil-Pirozzi et al. (2010) created a training program of internal memory strategies, including semantic association (categorization and clustering), semantic elaboration/chaining, and imagery. However, training methods included error-free learning with fading (O’Neil-Pirozzi et al. 2010). Perna and Perkey (2016) indicated that internal memory strategies “do not aim to salvage damaged brain tissue but to promote restoration of function” based on the idea that “impaired memory will respond to mental exercise” (p. 2). Strategies in the training included visualization, first letter mnemonics, putting words into sentences or stories, and semantic clustering (Perna and Perkey 2016). Across these studies, there is some mixture of the strategies and functions of the three approaches, most notably with internal memory aids included as part of the restoration approach.

The overlaps in function and strategies may be related to the development of the approaches throughout the years. Following the negative outcomes of restoration attempts with drill and practice based on the mental muscle idea, there was a shift to focus on alleviation of memory deficits and learning specific knowledge or skills relevant to a particular domain in life (Hunkin and Parkin 1995; Schacter and Glisky 1986). However, restoration and compensation were still considered as the two main approaches to rehabilitation, possibly due to similarities with the rehabilitation of other functions such as motor function or other cognitive functions such as attention (Cicerone et al. 2000; Kapur et al. 2002; Rees et al. 2007; Schacter and Glisky 1986). Thus, internal memory aids may have been incorporated into the restoration approach in place of drill and practice. While the approaches may be combined in rehabilitation for practical reasons, it is also essential to note that the different approaches require different training, materials, and provide different outcomes. For example, if the purpose of the rehabilitation is to provide daily functioning support, then training with external memory aids could be more appropriate. If, however, the purpose is to re-learn words, then knowledge acquisition strategies should be considered. Finally, the holistic approach may be the best placed to offer integration in community setting and for social participation. It is crucial to consider the compatibility of the rehabilitation goals with the selected memory rehabilitation approach.

## Future considerations

Based on the outcomes from previous studies, some considerations for future research arise. Firstly, consideration might be given to whether etiologies should be mixed or examined separately, followed by the severity, variety, and chronicity of memory impairment within the target group (Exner et al. 2021; Fetta et al. 2017; Kapur et al. 2004; Piras et al. 2011; Tate 1997). Individuals who are at different stages of recovery, with different etiologies, and capacities following memory impairment will require different amounts of training or practice with a given task to improve and will progress at different rates (Lexell et al. 2013; Vasquez et al. 2021). Thus, training duration is not only important to plan timings of the rehabilitation, but also it is important for each individual to have enough time for the rehabilitation to be effective (Leśniak et al. 2018; Vasquez et al. 2021). Secondly, memory needs in daily life should be assessed and matched to treatment approaches because improvements on laboratory tasks may not translate to functional improvements in daily life (Kapur et al. 2004; Perna and Perkey 2016; Wilson 2002). For example, while some individuals may not be able to implement errorless learning on their own, they may be able to quiz themselves, ask family members for help, or use an external aid to remember their grocery list when given appropriate training (Evans et al. 2020; Fish et al. 2015). Similarly, self-awareness and emotional disturbances may be severely impacted following ABI and future research should include self-awareness and emotional measures and disturbances in these domains should be addressed as part of treatment (Sansonetti et al. 2021; Terry et al. 2019; Urech et al. 2020; van Heugten and Wilson 2021). Moreover, it is evident that incorporating some aspect of improving self-awareness and psychoeducation as an aspect of rehabilitation such as feedback and involving family members can improve outcomes and should thus be considered regardless of the approach (Al Banna et al. 2015; Chouliara and Lincoln 2016; Long et al. 2014; Perna and Harik 2020; Sansonetti et al. 2021). Thus, self-awareness and emotional measures should be incorporated to assess current functioning levels and deficits in these domains should be addressed with education and feedback where possible.

Qualitative studies examining patient perspectives have demonstrated the importance of group settings for social interaction, feedback from others, and ability to compare progress in a motivational or appreciative capacity (Chouliara and Lincoln 2016; Leśniak et al. 2018; Lexell et al. 2013; Nilsson et al. 2011). In particular, with the rise of CACR options available within home settings, the group-based interactions found in traditional cognitive rehabilitation settings should not be entirely excluded, as social contact is likely a crucial aspect of rehabilitation (Pertíñez and Linares 2015; Withiel et al. 2020). In addition, goal setting and personalization of the treatment appear to be increasingly important factors in memory rehabilitation (Chouliara and Lincoln 2016; De Luca, et al. 2018a, b; Pertíñez and Linares 2015; Vasquez et al. 2021; Velikonja et al. 2014). It is essential to match the needs of the individual and their ability level with the appropriate rehabilitation approach and strategies. For example, some individuals may forego using external aids or electronic assistive devices with a preference for internal strategies if they prefer to not rely on external devices or they may need to try different strategies until finding the ones that are appropriate for their abilities and routines (Boman et al. 2010; Chouliara and Lincoln 2016; Nilsson et al. 2011). Finally, there is an increasing emphasis on the need to collect qualitative data from patients undergoing memory rehabilitation programs in order to better understand patient experiences and outcomes in addition to follow-up outcomes which may differ from the results obtained using objective measures (Chouliara and Lincoln 2016; Lexell et al. 2013; Miller and Radford 2014; Sarajuuri et al. 2018; Withiel et al. 2020). Thus, future research should include qualitative measures of patient perspectives to better adapt future rehabilitation programs to suit the needs of patients.

Finally, follow-up studies should be conducted to examine the lasting impact of memory rehabilitation. For example, recent studies have found that rehabilitation following ABI had positive impacts on community integration, quality of life, and continued use of external aids for several years beyond termination of the program (Shany-Ur et al. 2020; Svoboda et al. 2015). Similar follow-up studies would be beneficial for identifying which outcomes of memory rehabilitation are maintained long-term and generalize to functional outcomes. Thus far, memory rehabilitation has been approached through restoration of function, acquisition of specific knowledge, and compensation for memory failures. These approaches have shown some efficacy but require further examination individually and in combination to create memory rehabilitation programs that align with the needs of memory-impaired individuals. The introduction of the holistic approach has allowed for better results on psychological and emotional wellbeing factors associated with ABI but may require considerable resource and cost contributions. Several factors must be established when attempting memory rehabilitation, including the severity of the memory impairment, the needs of the patients in their everyday lives, the current memory abilities and self-awareness level of the patient, emotional disturbances, the objectives of the memory rehabilitation including the type of memory targeted, the compatibility of the stimuli and the strategies, and the potential for generalizability and long-term effects.

## Data Availability

Not applicable.
